# MEMS terahertz-to-infrared band converter using frequency selective planar metamaterial

**DOI:** 10.1038/s41598-018-30858-z

**Published:** 2018-08-20

**Authors:** Fabio Alves, Leroy Pimental, Dragoslav Grbovic, Gamani Karunasiri

**Affiliations:** Department of Physics, Naval Postgraduate School, Monterey, CA 93943 USA

## Abstract

A MEMS terahertz-to-infrared converter has been developed based on the unique properties of metamaterials that allow for selective control of the absorptivity and emissivity of the sensors. The converter consists of a sensing element structurally made of planar metamaterial membranes, connected to a substrate frame by four symmetrically-located thermal insulators. Upon THz absorption, the temperature of the sensing element increases and the outward infrared flux from the backside of the element is read by a commercial long-wave infrared camera. Two configurations were designed and fabricated with metamaterial absorptivity optimized for 3.8 THz and 4.75 THz quantum cascade lasers. The first sensor, fabricated with an oxidized aluminum backside, exhibits higher responsivity, but lower conversion efficiency than the second sensor, fabricated with a metamaterial backside. The spectral characteristics of the metamaterial on the two sides can be optimized to improve both responsivity and sensitivity, while keeping the sensors’ thermal time constant sufficiently small for real time imaging. No dedicated electronics or optics are required for readout making metamaterial-based MEMS THz-to-IR converters very attractive for THz imaging as means of a simple attachment to commercial IR cameras.

## Introduction

The realm of terahertz (THz) sensing evolved significantly in the recent years, particularly due to advances in metamaterials^1^. Relying on the ability to manipulate the optical properties of metamaterials using structural parameters, it has been possible to design efficient THz absorbers^2–6^ tuned to the frequency of interest for THz sensing^7–16^. In particular, microelectromechanical (MEMS) devices gained interest due to simplicity in fabrication and operation. Wen *et al*.^17^ demonstrated silicon nitride (SiN_x_)/gold (Au) MEMS meta-molecule arrays, fabricated on glass substrate, operating around 3 THz using cross-shaped metallic resonators with maximum absorptivity around 50%.

Bilgin *et al*.^18^ published his results on Parylene-C/titanium(Ti) MEMS sensors, operating from 0.5 to 2 THz, based on multi-dimensional metamaterial square elements. Our group^19,20^ has developed aluminum (Al)/silicon-rich silicon oxide (SiO_x_) metamaterial-based MEMS bi-material sensors to detect THz at 3.8 and 4.75 THz with absorptivities near 100%. In the detection scheme, employed by the above approaches, incident THz radiation, absorbed by a planar metamaterial structure, heats the attached bi-material beams that deform proportionally to the amount of absorbed THz radiation. Although it is simple to probe mechanical deformation using optical means, practical use of these sensors for imaging is somewhat challenging due to the complexity of optical readout. In addition, the free-standing movable structures are prone to residual stress that arises in the fabrication process. This increases the difficulties imposed on the optical readout setups, which are commonly employed with bi-material focal plane arrays (FPA).

One attractive possibility to overcome these limitations is reading the heat generated in each pixel of the FPA using a commercial infrared (IR) camera. In this context, there is no need for movable parts within the MEMS sensors, eliminating the problems associated with residual stress. Furthermore, since the sensor would perform a simple translation from THz-to-IR, no dedicated optics or electronics would be required. The idea of *band converters* is not new. Balageas *et al*.^21^ published a comprehensive review on photothermal visualization of electromagnetic fields using the heat-photon conversion phenomenon. According to Balageas, the first experimental demonstration was published in 1955 by Hasegawa^22^. It consisted of a filter paper coated by cobalt chloride (CoCl_2_). Cobalt chloride, while naturally pink, turns blue upon microwave absorption; however no quantitative information about field intensities was retrievable. Following several different schemes using polaroid films^23^, liquid crystals^24^, and photochromic films^25^, infrared thermography started to be used in 1975^26,27^ and matured in the early nineties with the use of radar-absorbing materials^28^. Essentially, an infrared camera is used to record the surface temperature of a continuous microwave absorbing film. Although different-imaging enhancement techniques have been studied^29,30^, sensitivity was limited by the films’ absorptivity, and spatial resolution was very poor. THz-to-NIR photon conversion was demonstrated by Nawata *et al*.^31^ using slant-strike periodically-poled lithium niobate (LiNbO_3_). While Nawata’s detection scheme was found to be very efficient, it was also very complex, and not suitable for real-time imaging. More recently, a pixel-less frequency up-conversion photon type terahertz imager has been demonstrated by Fu *et al*.^32^. In Fu’s device, gallium arsenide (GaAs), aluminum gallium arsenide (AlGaAs) and indium gallium arsenide (InGaAs) quantum wells are used to detect incoming THz radiation (intersubband transitions) and simultaneously emit NIR photons (interband transitions). Fu demonstrated detection frequencies between 4.5 to 7 THz and emission wavelengths between 880 and 920 μm. Despite the low cross-talk of the pixel-less structure, both, the low conversion efficiency and the low temperature required for operation impose some restrictions for practical devices.

Moldosanov *et al*.^33^ proposed a band converter based on metallic nanoparticles embedded in a THz-transparent matrix material. Partially filled electron density of states (DOS) at the Fermi energy of the nanoparticles assures absorption of the THz energy, converting it into a 2D pattern that can be visualized by an IR camera. Although Moldosonov claims fast response and sufficient sensitivity for passive THz imaging, he did not report any experimental demonstrations.

Pradere *et al*.^34^ demonstrated a photothermal converter for THz imaging based on thick carbon films on ceramic substrates. Pradere’s converter was tested using a commercial bolometric IR camera. The recorded maximum temperature increase was 1.6 °C for an incident flux of around 40 mW. The claimed minimum detectable THz power is on the order of 10 μW.

A much simpler band converter was proposed by Kuznetsov *et al*.^35^, for sub-terahertz (~0.3 THz) imaging using infrared camera. Kuznetsov’s metamaterial-based devices consist of an arrangement of sub-matrices with a periodic repetition of split ring resonators (SRR) on a polypropylene film backed by a metallic ground plane and an IR emissive layer. The “pixels” shared the same same dielectric and ground plane layer. Although no information was provided about the performance of the converters, it is reasonable to expect significant cross-talk (blooming effect) and polarization dependence absorption that could have affected the sensitivity. More recently, Fan *et al*.^36^ demonstrated all dielectric metasurface absorbers, based on etched silicon cylindrical resonators transferred to thin PDMS substrates that were used as an universal band converter. In particular, conversion was demonstrated from sub-terahertz (~600 GHz) to LWIR where the achieved responsivity was around 0.02 K/μW with a thermal time constant of 1000 ms. Despite of the simplicity of the devices, real time imaging would require significantly higher performance.

**Figure 1:**
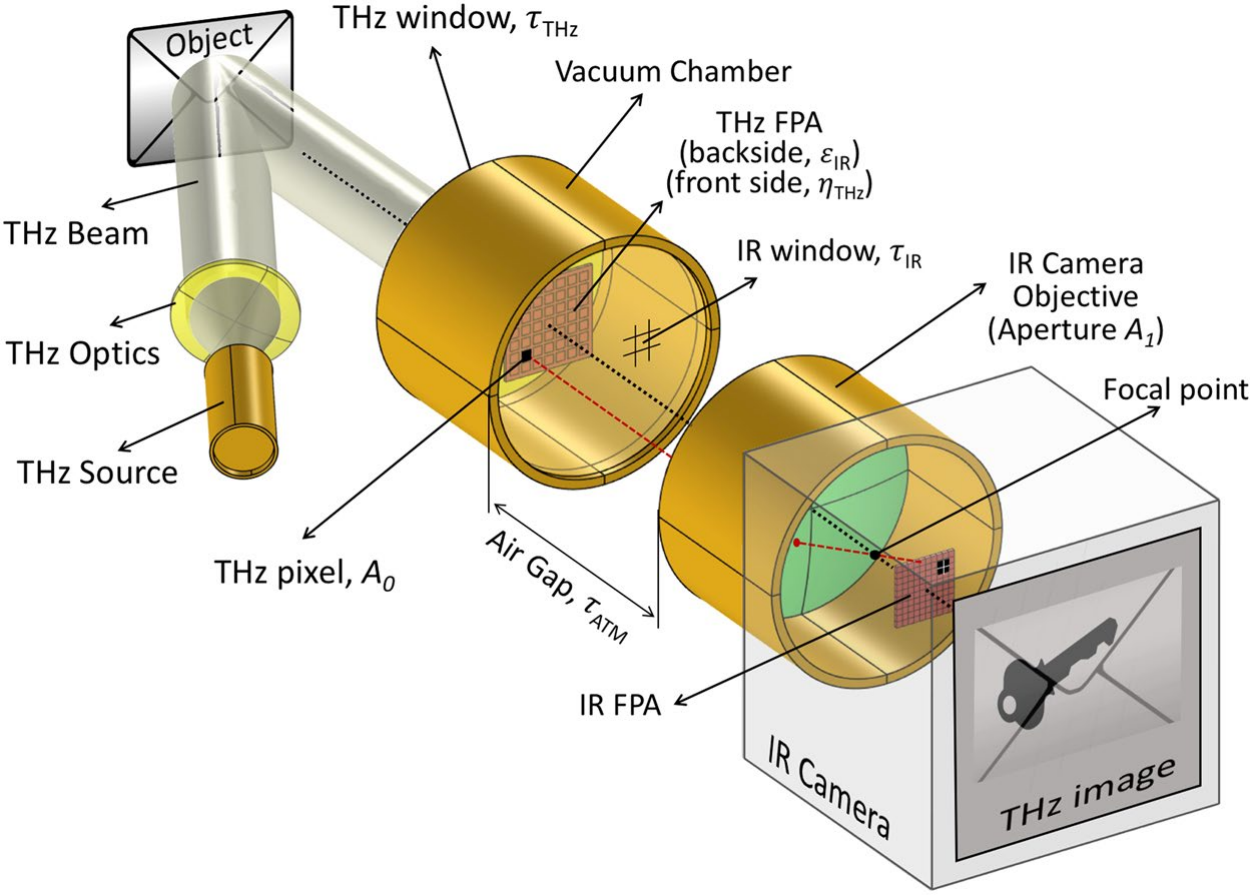
Terahertz to infrared imaging system concept. Schematic diagram of the imaging system from the illumination source (left) to the infrared camera (right). The transmissivity, absorptivity and emissivity of the components are represented by the Greek letters *τ*, *η* and *ε* with the appropriate indices. The THz-to-IR FPA is placed inside a vacuum chamber with a THz-transparent window (receiving side) and a IR-transparent window (emitting side). The THz pixel area is represented by *A*_*0*_ while the IR camera optical aperture area is represented by *A*_*1*_. The object is depicted as an envelope with a concealed metallic key inside, showing in the THz image. Each THz pixel (dark square on the THz FPA) is mapped on a subarray of pixels on the IR camera FPA (follow the dashed red line).

The detection scheme proposed in this paper builds on Kuznetsov’s idea. It uses planar metamaterial structures as highly efficient absorbers in the THz range of interest, as well as selective emitters^37^, optimized for the IR spectral band of the readout camera. The band selectivity is important for increasing the conversion efficiency. We propose the use of MEMS focal plane arrays with individually insulated membranes (pixels) with double-side, uncoupled, planar metamaterials. Figure 1 shows a schematic diagram of the THz-to-IR imaging system, highlighting the details and properties of components that will be considered in the analysis to follow.

In Fig. 1, an arbitrary object is illuminated by a THz source and the reflected radiation is captured by the MEMS THz-to-IR FPA. The FPA is sitting inside a vacuum chamber with two different windows. On the THz side, a window with transmissivity *τ*_*THz*_ allows most of the THz radiation to reach the sensor, while on the IR side a window with transmissivity *τ*_*IR*_ allows most of the IR flux to be transmitted. Each pixel of the FPA, whose area is *A*_*0*_, absorbs THz with absorptivity *η*_*THz*_ ≡ *ε*_*THz*_ on the front-side, and emits IR with emissivity *ε*_*IR*_ on the backside. The air between the sensor chamber and the IR camera has transmissivity of *τ*_*ATM*_. The optical aperture of the IR camera has an area of *A*_*1*_, while the transmissivity of the objective and the spectral filters, as well as the IR sensor spectral sensitivity are lumped into a single parameter, *τ*_*IR-Camera*_.

It is worth mentioning that the thermal background has less energy in the THz spectral range, illumination sources are often required for THz imaging. Currently, the most powerful sources for frequencies higher than 3 THz are quantum cascade lasers (QCL)^38–40^, not considering free electron lasers (FEL)^41^, which are not suitable for practical applications.

## MEMS THz-to-IR Thermal Sensor

**Figure 2:**
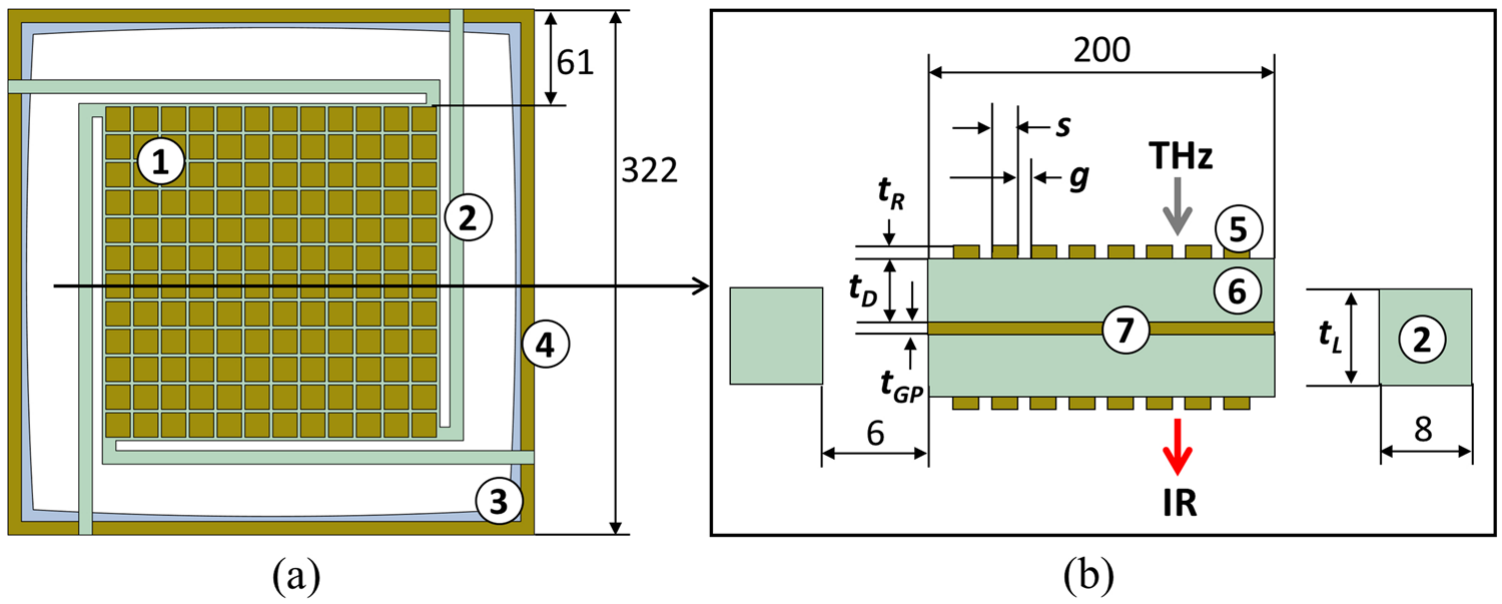
Metamaterial-based MEMS THz-to-IR band converter. (**a**) Top-view layout of a single pixel device showing the planar metamaterial absorber (1), held in place by four symmetric thermal insulating beams (2) connected to the substrate frame (3). An aluminum frame (4) acts as a heatsink. In a focal plane array, this pixel repeats in a 2D fashion. (**b**) Cross sectional layout of the sensor depicted in (**a**) showing two identical planar metamaterial structures mirrored around the ground plane, where the aluminum resonators (5) are sitting on a dielectric spacer (6). The ground plane (7) works for both metamaterial structures. All dimensions are in μm and *t*_*D*_, *t*_*R*_, *t*_*GP*_, *t*_*L*_ are the thicknesses of the dielectric, resonators, ground plane and thermal insulating beams, respectively. The resonators size and gap between them are represented by *s* and *g*, respectively.

Efficient THz-to-IR converters should exhibit high absorptivity in the detection band of interest as well as highly-selective emissivity in the band of the readout infrared camera. This can be achieved with planar metamaterials. The structures used for the proposed sensors are simple periodic repetitions of metallic elements (resonators), separated from a homogeneous metallic layer (ground plane) by a dielectric spacer^5,19,20^. The spectral response of the absorptivity/emissivity can be controlled by selecting the geometric configuration and material properties. Figure 2 shows a schematic diagram, front (top view) and cross section, of the proposed metamaterial-based MEMS THz-to-IR band converter.

A single sensor is comprised of a double planar metamaterial structure, connected to the array frame (substrate) by symmetrically located thermal insulating beams. As the front-side metamaterial structure absorbs the incoming THz radiation, the temperature increases from *T*_*0*_ (ambient temperature) to *T*_*0*_ + *ΔT* and the backside metamaterial structure selectively radiates into the infrared band of interest.

In the sensing element, the effect of the absorbed THz flux is twofold. There is change in the detector element temperature, as heat is lost through conduction via the thermal insulator beams and radiation on both sides of the element. Heat loss through convection can be neglected since most of thermal sensors operate inside vacuum packages. Both heat loss mechanisms can be lumped into a single parameter, thermal conductance *G*_*th*_. Thus, the temperature difference in the detector can be evaluated by solving the heat balance equation:

1 $${C}_{th}\frac{d({\rm{\Delta }}T)}{dt}+{G}_{th}{\rm{\Delta }}T={\eta }_{THz}{{\rm{\Delta }}{\rm{\Phi }}}_{0},$$

where *ΔΦ*_*0*_ is the change in the incident flux, *η*_*THz*_ is the sensing element absorptivity, which is the fraction of the incident flux absorbed by the front-side metamaterial layer, and *C*_*th*_ is the thermal capacitance of the sensing element. While *C*_*th*_ [J/K] depends on the heat capacity and mass of the constituent materials of the detector element, *G*_*th*_ [W/K] is the derivative of the outgoing flux with respect to the temperature. The two thermal constants can be calculated using the following expressions:

2 $${C}_{th}=\sum _{N}\,{c}_{th}\rho {A}_{0}d$$

and

3 $${G}_{th}=\frac{d({{\rm{\Phi }}}_{\nu R}+{{\rm{\Phi }}}_{C})}{dT},$$

where the *N* is the number of different material layers of the sensing element and *c*_*th*_, ρ and *d* are the thermal capacity, density and thickness of each layer, respectively, and *A*_*0*_ is the total surface area of the sensing element. The conductive flux, *Φ*_*C*_, associated with the thermal insulating beams is obtained by:

4 $${{\rm{\Phi }}}_{C}=4\frac{{\kappa }_{th}{A}_{C}{\rm{\Delta }}T}{{L}_{leg}},$$

where *κ*_*th*_ is the thermal conductivity of the material, *A*_*c*_ represents the cross-sectional area of the beam, *L*_*leg*_ is the net linear dimension and *ΔT* is the temperature difference between the sensing element and the array frame (substrate). The substrate mass is much larger than that of the sensing element. Therefore, it is considered a heat sink at ambient temperature *T*_*0*_. The multiplication by four in Eq. 4 accounts for four beams (see Fig. 2).

The radiative flux, *Φ*_*νR*_ [W], is treated here as a radiometric quantity, defined as the amount of optical power flowing into or out of a surface. The total radiative flux is the sum of the radiative fluxes on both sides of the sensor and it can be represented by:

5 $${{\rm{\Phi }}}_{\nu R}={A}_{0}\pi {\int }_{0}^{\infty }\,{\varepsilon }_{THz}(\nu ){L}_{0\nu }d\nu +{A}_{0}\pi {\int }_{0}^{\infty }\,{\varepsilon }_{IR}(\nu ){L}_{0\nu }d\nu ,$$

where *ε*_*IR*_ and *ε*_*THz*_ are the emissivity/absorptivity of the metamaterial layers on the backside and front-side of the sensing element respectively. The spectral radiance, *L*_0*ν*_ [W/(m^2^sr)], is given by:

6 $${L}_{0\nu }=(\frac{2h{\nu }^{3}}{{c}^{2}})\frac{1}{\exp (\frac{h\nu }{kT})-1},$$

where *h* = 6.62 × 10^−34^ [m^2^ kg/s] is the Planck constant, *c* = 2.9979 × 10^8^ [m/s] is the speed of light in vacuum, *k* = 1.38 × 10^−23^ [m^2^ kg/(s^2^ K)] is the Boltzmann constant. The temperature *T* is the absolute temperature of the sensing element (*T* = *T*_*0*_ + *ΔT*).

The solution of Eq. 1, assuming a periodic incident *ΔΦ*_*0*_, is given by:

7 $${\rm{\Delta }}T=\frac{{\eta }_{THz}{{\rm{\Delta }}{\rm{\Phi }}}_{0}}{{G}_{th}\sqrt{1+{(\omega {\tau }_{th})}^{2}}},$$

where *ω* is the angular frequency of *ΔΦ*_*0*_ and *τ*_*th*_ = *C*_*th*_/*G*_*th*_ is the thermal time constant of the sensor. For *ω* ≪ *1/τ*_*th*_, *ΔT* ≈ *η*_*THz*_*ΔΦ*_*0*_/*G*_*th*_. Two important figures of merit of thermal sensors are first, the speed of operation, defined by the thermal time constant (*f*_*OP*_ = 1/(2*πτ*_*th*_) [Hz]) and second, responsivity, which is the temperature difference per unit incident flux difference (*R* = *ΔT*/*ΔΦ*_*0*_ = *η*_*THz*_*/G*_*th*_ [K/μW]). In order to maximize responsivity, the metamaterial absorptivity in the band of interest has to be maximized, while thermal conductance has to be minimized. This, however, directly impacts the thermal time constant, which is inversely proportional to *G*_*th*_, making the detector operate at a slower speed. To compensate, a reduction in thermal capacitance is required. However, since this parameter depends on device dimensions/materials, this can directly affect the metamaterial absorption^5,42^. All parameters are coupled and the nature of the application will define the compromise to be made in the sensor design.

It is important to notice that increasing the responsivity of the sensor does not necessarily increase the performance of the system. For example, if the incident flux (*ΔΦ*_*0*_) is kept constant and the emissivity of the backside of the sensor is reduced, the outward radiative flux will also be reduced. The temperature rise is not sufficient to increase the radiative flux back to the previous level because of the increase of conductive heat flux through the anchoring beams. At steady state, even though the responsivity (*ΔT*/*ΔΦ*_*0*_) will increase, the backside radiative flux will decrease.

Because the IR camera senses the radiative flux, reducing the backside emissivity of the sensing element reduces the overall performance of the system, even though the responsivity of the sensing element increases. For design purposes, it is useful to define the conversion efficiency, which in this particular case will be considered to be the ratio between the change in the flux radiated by the backside of the sensor (*ΔΦν*_*IR*_) within the spectral band of the chosen IR camera and the total incident flux change on the front-side of the sensor (*ΔΦ*_*0*_). Assuming the sensing element is a Lambertian radiator^43^, the intrinsic efficiency of the sensor can be mathematically represented as:

8 $$eff=\frac{{A}_{0}\pi ({\int }_{{\nu }_{1}}^{{\nu }_{2}}\,{\varepsilon }_{IR}(\nu ){L}_{0\nu }(T)d\nu -{\int }_{{\nu }_{1}}^{{\nu }_{2}}\,{\varepsilon }_{IR}(\nu ){L}_{0\nu }({T}_{0})d\nu )}{{{\rm{\Delta }}{\rm{\Phi }}}_{0}}$$

where *A*_*0*_ is the area of the sensing element, the multiplicative *π* outside the integral is the hemispherical projected solid angle [sr], *ε*_*IR*_ is the emissivity of the backside of the sensor, and the integration limits are the frequency limits of the infrared camera spectral response. Since *ΔΦ*_*0*_ = *ΔT*/*R*, *eff* = *ΔΦν*_*IR*_ × *R*/*ΔT*, which relates the outward heat flux difference from the backside of the sensing element in the spectral band of the IR camera with responsivity, better representing the performance of the THz-to-IR converter.

**Figure 3:**
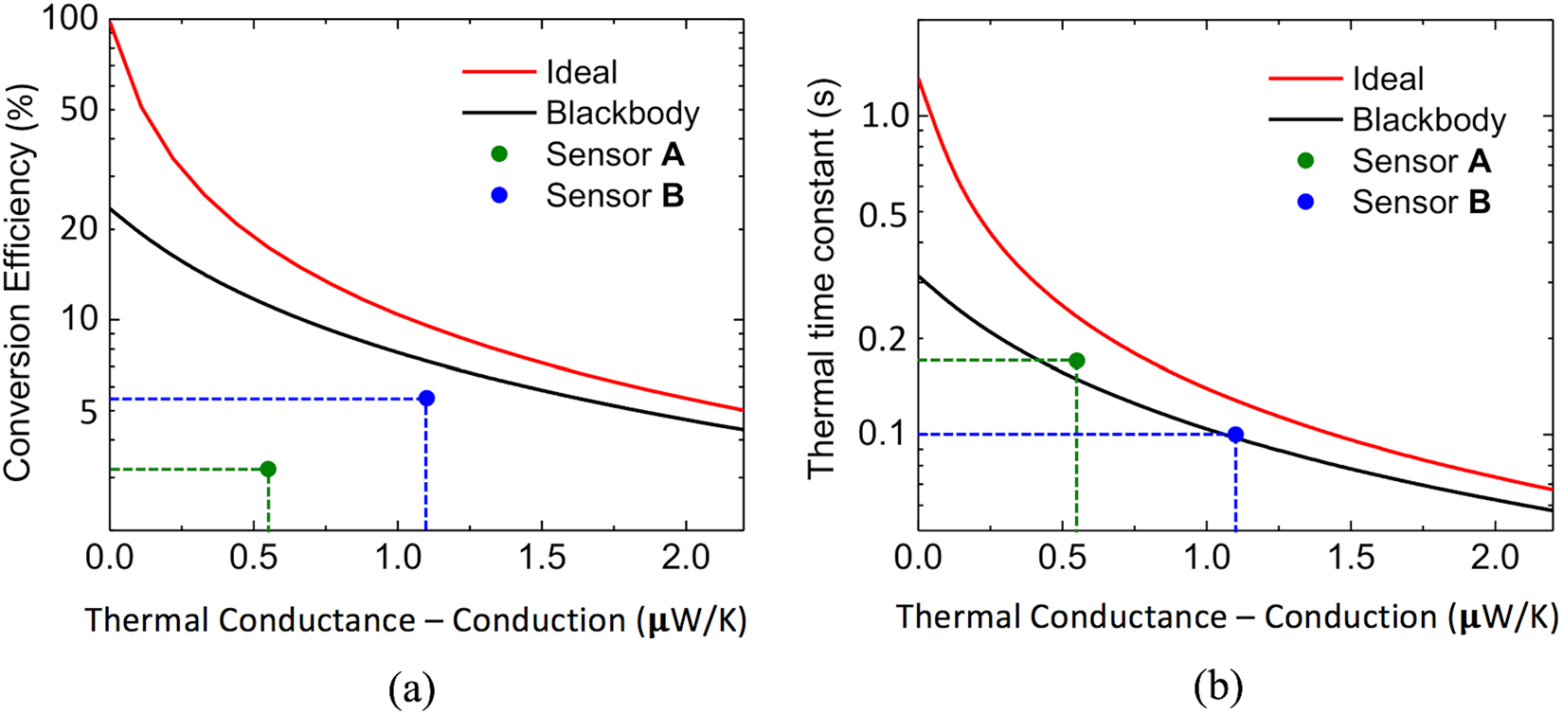
Efficiency and Thermal time constant of the THz to IR sensor. Effect of the thermal conductance via the beams, *G*_*Cond*_, on conversion efficiency (**a**) and thermal time constant (**b**) when the sensing element surfaces are ideally selective (red lines) and perfect blackbodies (black lines). The markers indicate the performances of sensor A and B (see the Design section).

Efficiency can be increased by reducing the thermal conductance due to conduction through the thermal insulator beams and by finely tuning the metamaterials to the spectral bands of interest. The fundamental limit would be reached when absorptivity of the front side metamaterial is equal to 1 (100%) at the band of the THz source and zero otherwise and when emissivity of the backside metamaterial is equal to 1 at the band of the IR camera and zero otherwise (ideally selective). Figure 3(a,b) show how the conversion efficiency and thermal time constant, respectively of the proposed sensor varies with thermal conductance via the beams (*G*_*Cond*_) when the absorptivity/emissivity are ideally selective (red line) and when both sides of the sensing element act as blackbodies (*ε*_*IR*_ = *ε*_*THz*_ = 1) (black line). The computation was performed for room temperature operation, *T*_*0*_ = 300 K, *ΔT* = 1 K and sensing element area *A*_*0*_ = 200 μm × 200 μm. The markers represent the performance parameters of sensors A and B, to be described in the next section.

Thermal conductance via the beams can be controlled by the geometry and material of the thermal insulator beams, respecting fabrication and structural limits. As *G*_*Cond*_ increases, conduction becomes the dominant mechanism of outward heat flux, reducing the effects of the radiation selective surfaces on the performance parameters.

## Design

**Figure 4:**
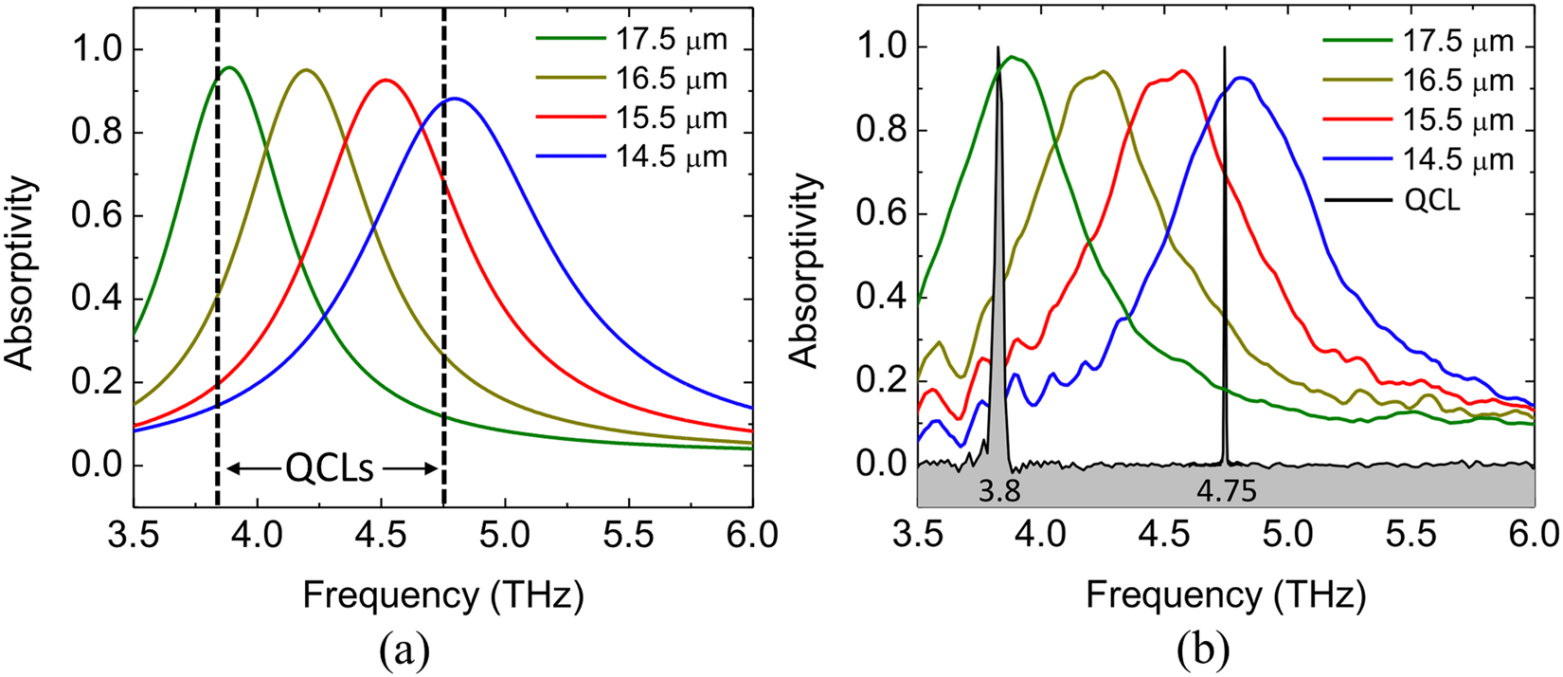
THz planar metamaterial frequency response. Simulated (**a**) and measured (**b**) absorptivity (colored lines) of four planar metamaterial surfaces with different resonator sizes. The black lines represent the normalized measured QCL responses at 3.8 and 4.75 THz. Notice that structures with resonator sizes of 17.5 and 14.5 μm are well matched with the 3.8 and 4.75 THz, respectively.

The THz-to-IR sensors were designed through finite element modeling using the commercial software COMSOL Multiphysics^44^, due to the software’s capability to simultaneously handle multiple phenomena. The periodic nature of the planar metamaterials allows for simulation to be conducted on a unit cell basis. The full electromagnetic field simulation was performed using a 2-port, 3D configuration where a plane wave from port 1 passes through the metamaterial unit cell. Due to the presence of the ground plane in the structures, whose thickness is greater than the skin depth for the frequencies of interest, there is no transmission. Periodic boundary conditions assure the higher-order scattering from the surrounding cells will be captured by port 1; therefore, absorptivity/emissivity of the structure becomes a direct reading of 1 − |*S*_*11*_|^2^ (see the Methods’ section for details). Several different Al/SiO_x_ planar metamaterial structures were simulated and fabricated using standard MEMS microfabrication techniques^45^ Fig. 4 shows the simulated responses of four different structures, compared with the measurements, highlighting the accuracy of the models. The measurement techniques are discussed in Methods.

**Figure 5:**
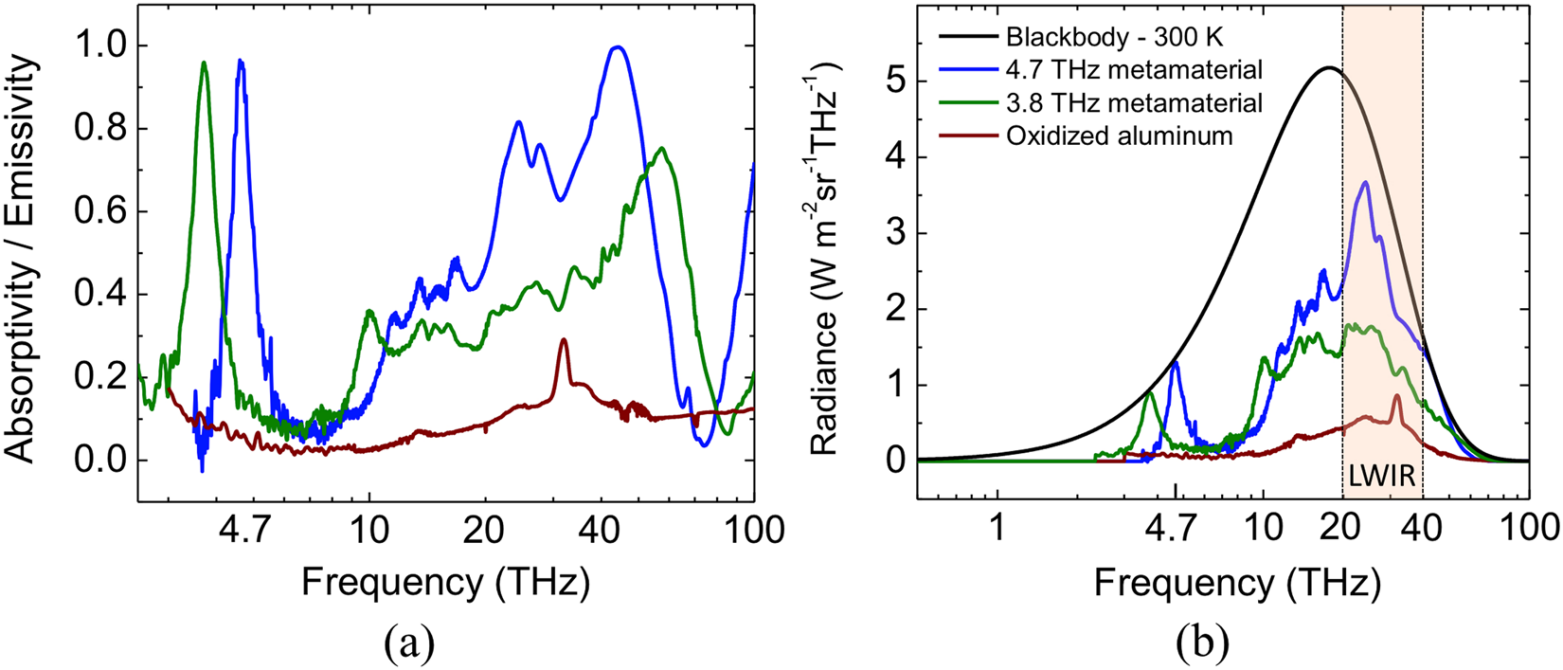
Measured emissivities and estimated radiances of sensors A and B front and backside. (**a**) Measured emissivities of the surfaces for sensor A front side (3.8 THz metamaterial), sensor A backside (oxidized Al) and sensor B front and backside (4.75 THz metamaterial) from THz to MWIR. (**b**) Estimated radiance of all surfaces compared with a blackbody radiance. The shaded region represents the LWIR camera sensitivity band (from 20 to 40 THz). The legends in (**a**) are the same as those in (**b**).

Metamaterial structures with peak resonance at 3.8 and 4.75 THz, that match with the quantum cascade lasers (QCL) available in our laboratory (see Fig. 4(b)), were selected to be incorporated into the sensors. Both show absorptivity/emissivity around 0.9 at the frequency of the THz lasers. As a proof-of-concept, two different sensor configurations were designed, both using SiO_x_ and Al on a Si substrate (frame). The first sensor (sensor A) had the front side tuned to the 3.8 THz QCL and the backside comprised of oxidized aluminum. The second sensor (sensor B) had the front side tuned to the 4.75 THz sensor and the backside comprised of the same metamaterial structure as on the front side, because it was found to exhibit selective emissivity in the LWIR band (see Fig. 5). Table 1 summarizes the geometrical parameters of the two sensors.

**Table 1:** Geometrical parameters of sensors A and B (see Fig. 2).

Sensor	*Front side*	*Backside*	*t*_*D*_ (μm)	*t*_*R*_ (nm)	*t*_*GP*_ (nm)	*t*_*L*_ (μm)	*s* (μm)	*g* (μm)
A (3.8 THz)	meta.	oxid. Al	1.1	220	220	1.1	17.5	2
B (4.75 THz)	meta.	meta.	1.1	90	90	2.2	14.5	2

Figure 5(a) shows the measured spectral responses of both metamaterial structures (sensor A and B) from THz to MWIR, as well as the oxidized aluminum. Figure 5(b) shows a comparison between the spectral radiance weighed by the surface emissivities shown in Fig. 5(a), and a blackbody radiance at 300 K.

As it can be seen in Fig. 5(a), the metamaterial structures optimized for THz absorption also show interesting emission characteristics in the LWIR range, which are far from ideal, but still suitable for a proof-of-concept. The ability to use the same metamaterial structure for the front and backside of the sensing element of sensor B significantly simplifies the fabrication as the same photolithographic mask can be utilized. The conversion efficiency for both configurations (A and B) were estimated using eqs (5–8) and plotted in Fig. 3. It is clear that the metamaterial selective emitter exhibits higher efficiency, and a smaller thermal time constant than the oxidized aluminum (see Fig. 3).

The characteristics of sensors A and B single pixels were extensively simulated in COMSOL. The absorbed power by the pixel was simulated as an incoming heat flux, distributed over the front surface area of the sensing element. The amount of heat flux was set as the absorptivity (*η*_*s*_) at the QCL frequency (3.8 or 4.75 THz) multiplied by 1 μW; therefore, all calculations would be normalized by 1 μW incident power. Material properties were extracted from the literature and are summarized in Table 2.

**Table 2:** Properties of the constituent materials of the MEMS THz-to-IR sensors^48,51,52^.

Material	*Young’s Modulus* *E* (×10^6^ *Pa*)	*Expansion Coefficient* *α* (×10^−6^ *K*^−1^)	*Thermal Conductivity* *g* (*Wm*^−1^ *K*^−1^)	*Heat Capacity* *c* (*J kg*^−1^ *K*^−1^)	*Density* *ρ* (*g m*^−3^)	*Electric Conductivity* *σ* (×10^6^ *S m*^−1^)	*THz refractive index* *n*
Si	100	2.7	130	750	2330	—	3.48–0.01i
SiO_2_	68	0.4	1.4	703	2200	—	2.0–0.02i
Al	70	25	237	900	2700	10	—

The non-stoichiometric composition of SiO_x_ has minimal impact in most properties except thermal conductivity, which was found to be around 4 W/(m K) due to the higher concentration of Si. This value was obtained by fitting the measured thermal response of the sensors with Eq. (3).

Another important figure-of-merit is noise, which for this detection scheme (Fig. 1) arises from different sources such as fluctuations in sensor temperature, background temperature, and illumination sources, as well as the readout system, and thermo-mechanical oscillations^46^. In practice, the total noise of the system is given by the noise equivalent power (NEP), which in our particular case can be defined as the incident radiant flux that produces the minimum detectable temperature difference by the IR camera used in the system (noise equivalent temperature difference – NETD). This is only valid if the sensor’s NETD is less than or equal to the camera’s NETD. In this case, the detection system is limited by the characteristics of the readout. Typically, high-end IR cameras exhibit NETDs between 10 and 100 mK. In order to verify the limiting factors of this detection scheme, total noise (*Φ*_*N*_) of the sensor can be estimated using^47^:

9 $${{\rm{\Phi }}}_{N}=\,\sqrt{(8\sigma {k}_{B}{T}^{5}{A}_{BB}B)+(4{k}_{B}{G}_{Cond}{T}^{2}B)},$$

where σ = 5.67 × 10^−8^ [W/(m^2^ K^4^)] is the Stephan-Boltzmann constant, *A*_*BB*_ is the area of the system aperture on the THz side (THz window in Fig. 1), and *B* is the bandwidth, which depends on the thermal time constant of the sensors. The first term within the parenthesis represents the fluctuations of temperature due to heat exchange between the sensor and its background via radiation. This component has the maximum value when absorption and emission elements behave as blackbodies. The second term represents temperature fluctuations of the sensing element via the beams where *G*_*Cond*_ is the associated thermal conductance (derivative of eq. (4) with respect to temperature difference, *ΔT*). Thermomechanical noise and instabilities of the illumination source were neglected since their contributions are relatively small^40,48^. The sensor NETD can be calculated using the sensor responsivity, assuming there is no initial incident flux, thus *ΔΦ*_*0*_ = *Φ*_*N*_ and the sensor *NETD* = *η*_*THz*_*Φ*_*N*_/*G*_*th*_. The NETDs of both sensors, A and B, were calculated, and are listed in Table 3. The values are around one order of magnitude lower than the IR camera; therefore, the noise of the imaging system is limited by NETD of the IR Camera. All design estimations are presented in Table 3 together with the experimental results.

**Table 3:** Estimated and measured parameters of the fabricated THz-to-IR sensors.

Sensor	A		B	
Property	Estimation	Measurement	Estimation	Measurement
*η*_*THz*_	0.93	0.95	0.85	0.9
*ε*_*THz*_	—	Fig. 5(a)	—	Fig. 5(a)
*ε*_*IR*_	—	Fig. 5(a)	—	Fig. 5(a)
*G*_*Cond*_ (μW/K)	0.55	—	1.10	—
*G*_*Rad*_ (μW/K)	0.02	—	0.30	—
*G*_*Total*_ (μW/K)	0.57	—	1.40	—
*C*_*th*_ ((μJ/K)	0.10	—	0.16	—
*τ*_*th*_ (m_s_)	180	170	110	100
*R* (K/μW)	1.7	1.75	0.64	0.66
*eff* (%)	3.2	—	5.5	—
NETD (μK)	150	—	70	—

## Experimental Results

**Figure 6:**
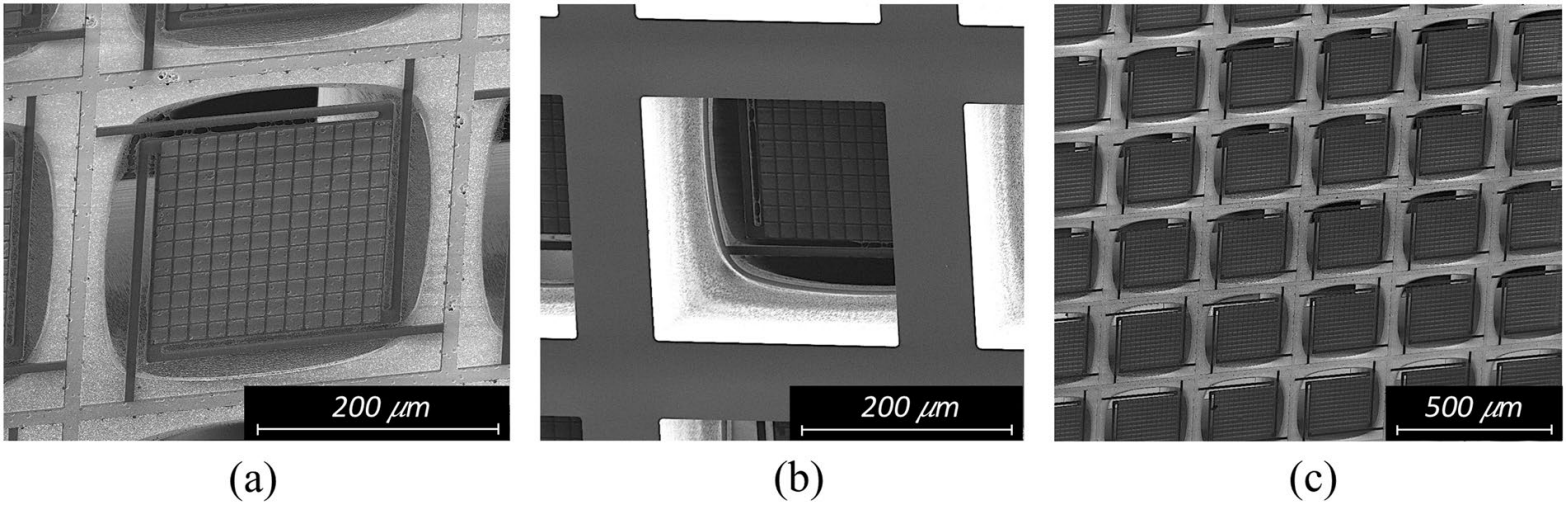
MEMS metamaterial-based THz-to-IR sensor SEM micrographs. SEM micrographs of fabricated sensor B (**a**) front side, (**b**) backside and (**c**) a section of the focal plane array. Images were made using beam excitation of 2 kV and magnification of 700X, 650X and 400X respectively.

Both sensors A and B were fabricated using standard MEMS microfabrication techniques (see Methods). Figure 6 shows the scanning electron microscope (SEM) micrograph of the front and back sides of sensor B, and a partial view of the FPA (front).

For the emissivity measurement, both sensors were attached to a heater and their temperature raised around 10 K above ambient. A FLIR-LWIR camera T1030sc was focused on the back side of the sensors. After thermal equilibrium was achieved, the emissivity in the camera settings was adjusted to allow the measured temperature to match the actual temperature of the heater. The recorded emissivities were used to tune the camera to each sensor individually.

**Figure 7:**
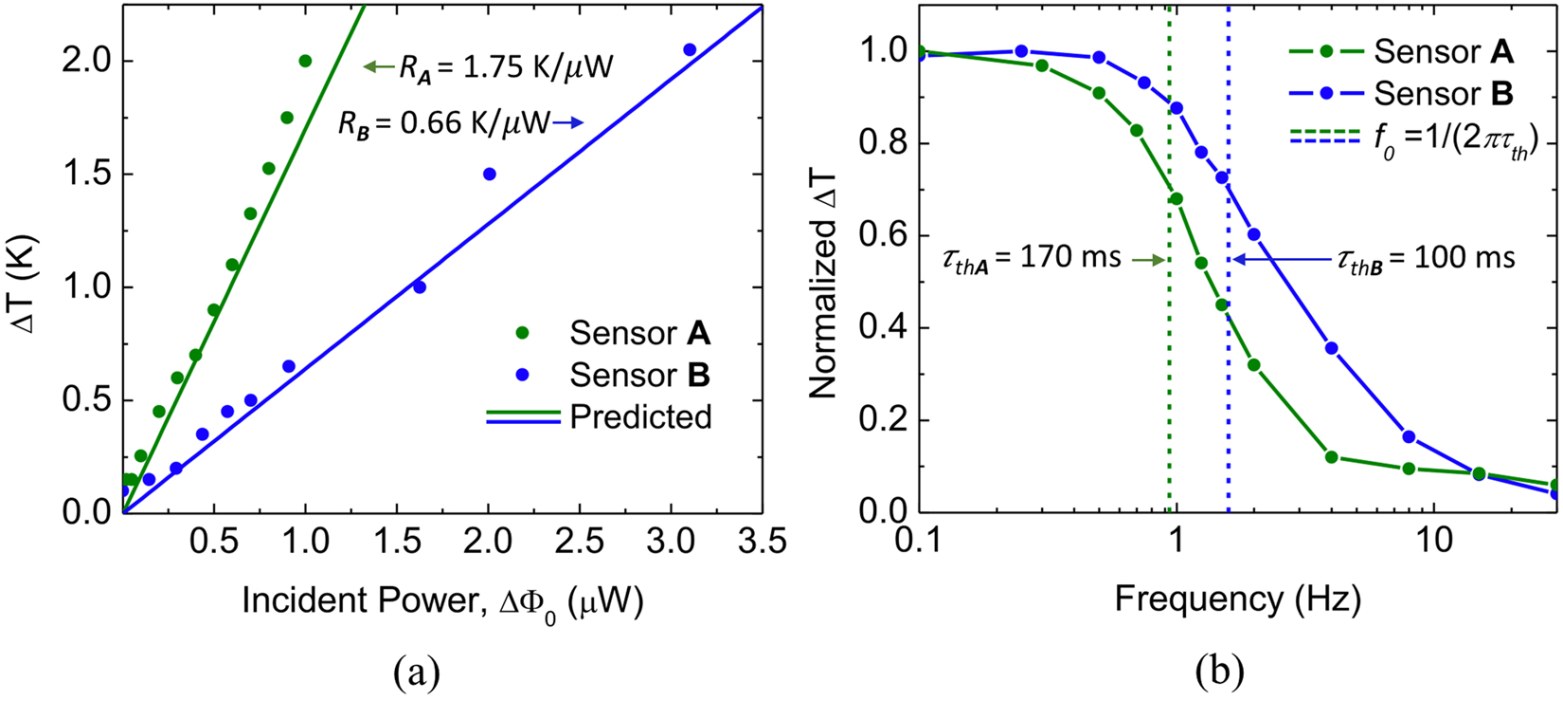
MEMS metamaterial-based THz-to-IR sensors figures of merit. (**a**) Estimated (solid line) and measured (solid dots) sensitivity of sensors A and B. Responsivity is given by the slope of the lines. (**b**) Measured frequency response of sensors A and B. The dashed lines indicate the cutoff frequency (*f*_0_), previously defined as frequency of operation (*f*_*OP*_) and thermal time constants are obtained by *τ*_*th*_ = 1/(2*πf*_0_).

The sensors were placed in a vacuum chamber with two optical windows on opposite sides. The THz side consisted of a 2 mm thick Tsurupica window, which transmits about 70% of the THz radiation of the QCL and blocks the IR bands^37^. On the IR side, a 2 mm thick zinc selenide (ZnSe) window, which transmits about 90% in the LWIR, was employed. The FLIR T1030sc camera was used to measure the heat flux, and to record the temperature of the backside of the THz-to-IR FPA. Two QCLs, 3.8 and 4.75 THz, were used to illuminate the corresponding sensors. Responsivity was obtained by varying the incident power levels and reading *ΔT* at individual pixels. The incident THz power on sensors A and B was measured using a calibrated THz power meter (see Methods), placed at the same position as the sensors under test, preceded by the Tsurupica window. Figure 7(a) shows a comparison between the measured data points (solid dots), and the simulated responses (solid lines) for each sensor.

**Figure 8:**
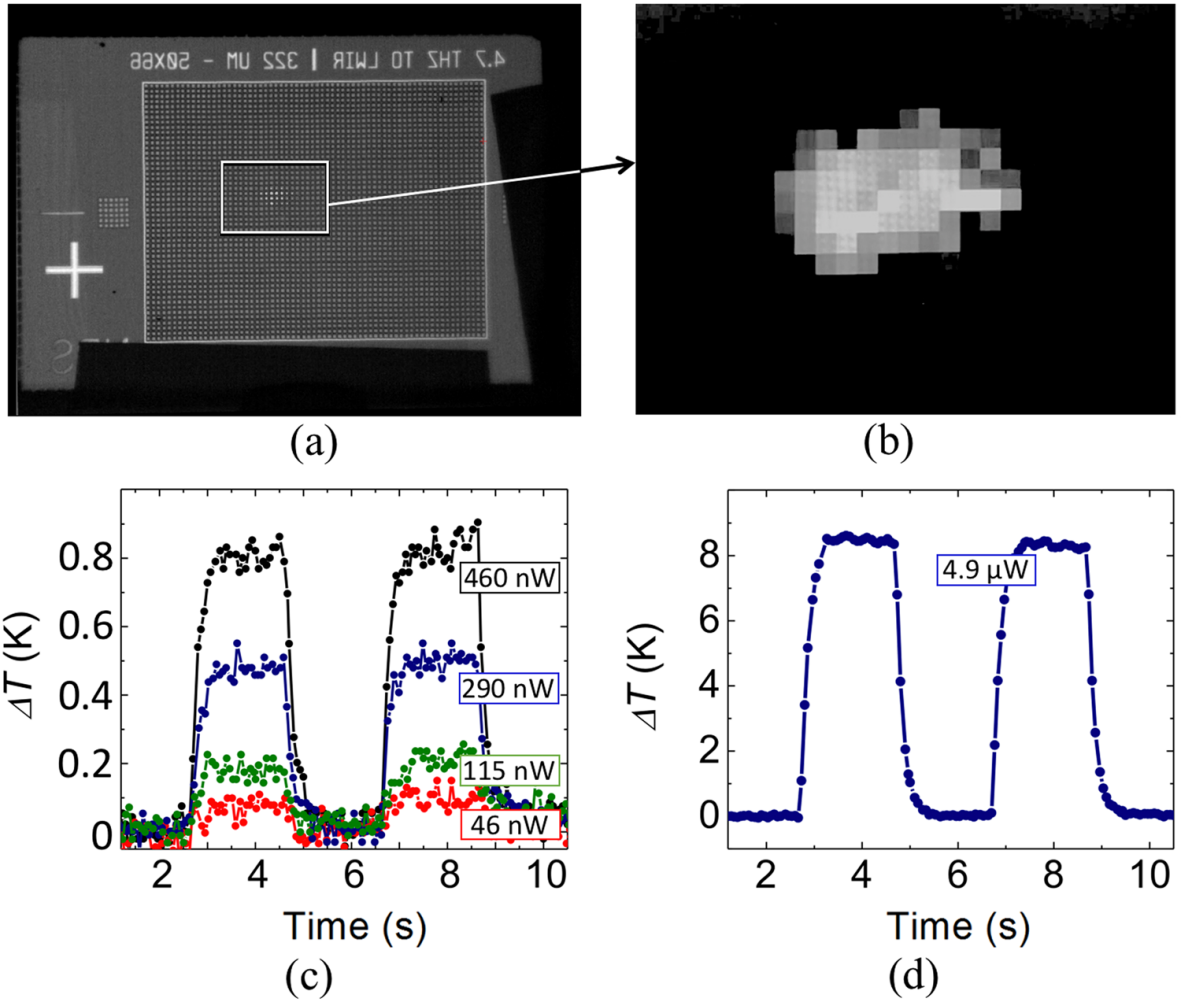
Image of the THz QCL taken by sensor B FPA. (**a**) Raw infrared snapshot of the backside of FPA with sensor B pixels showing in the center an elliptical 4.75 THz QCL beam. (**b**) Zoom in of the QCL image (white frame in (**a**)) after background subtraction and pixel filling. (**c**) Temporal response of the central pixel for a QCL is gated at 250 mHz for different incident power levels. Notice that *ΔT* < 80 mK can still be easily distinguished. (**d**) Temporal response when the incident power is 4.9 μW, highlighting the exponential temperature rise and fall due to the thermal time constant.

The responsivities were found to be 1.75 K/μW and 0.66 K/μW for the sensors A and B, respectively, and agree well with the predictions. Sensor B, with a metamaterial-based backside emitter, loses more heat through radiation than sensor A, with its oxidized aluminum emitter on the backside. NETD was measured by reducing the incident power of the QCLs until the temperature difference recorded by the camera was undistinguishable. For both sensors this value coincides with the FLIR camera NETD (~40 mK). The frequency responses of the two sensors were measured by modulating the QCLs at frequencies from 100 mHz to 30 Hz, and measuring the peak-to-peak temperature difference using the infrared camera. Figure 6(b) shows the measured frequency responses. The thermal time constants estimated by *τ*_*th*_ = 1/(2*πf*_*0*_) were found to be 170 and 100 ms for sensors A and B, respectively. Since the readout is simultaneously performed for all pixels, focal plane arrays of sensors A and B can operate at 6 and 10 FPS, respectively. Since *τ*_*th*_ = *C*_*th*_/*G*_*th*_, the only way to make the sensors faster without compromising responsivity is to reduce their thermal capacitance. This is not straightforward, since any change in material dimensions can directly affect the metamaterial optical properties, therefore reducing *η*_*THz*_^42^. Although the fabricated THz FPA is far from optimal, images of an ellipsoidal shaped 4.75 THz QCL beam pulsing at 1 Hz, taken using sensor B were recorded using the FLIR ResearchIR software. The raw snapshot of the FPA backside showing the QCL beam is shown in Fig. 8(a). Figure 8(b) shows a zoom in of the central area highlighting the imaged QCL beam after simple background subtraction and pixel filling, which was achieved by a simple algorithm that fills the whole area of the pixel with the recorded average temperature, and eliminates the dark areas between pixels.

Although the beam cannot be precisely resolved, its ellipsoidal shape is visible. The temperature difference in the central pixel was recorded by different incident power levels and showed in Fig. 8(c,d), which reveals that incident power lower of than 50 nW (*ΔT* < 80 mK) can be easily resolved just by graph inspection. The minimum detectable *ΔT* is about one half of that (~40 mK).

The primary readout approaches used by current THz thermal imagers are bi-material, bolometric or THz-to-IR conversion with the help of an IR camera. The cost of these schemes can be similar, due to the availability of relatively inexpensive microbolometer cameras with good sensitivity. Based on the published data on different THz imaging schemes, the performance is limited mainly by the readout and measured NETD values vary from 40–150 mK^19,49^. The imaging system NETD reported in this work lies at the low end of this range, based on specs of the microbolometer camera employed, making the performance of the reported configuration comparable with other detection schemes.

## Discussion

Driven by the recent interest in real time THz imaging, a THz-to-IR converter focal plane array using planar metamaterial structures was developed. New concepts were introduced to enhance efficiency of the conversion by tuning THz absorptivity at the frequency of the illuminating THz source, and the IR emissivity within the band of the infrared readout. Sensors comprised of metamaterial perfect absorbers with peak absorptivity at 3.8 and 4.75 THz QCLs were fabricated in focal plane array format. Oxidized aluminum and planar metamaterial were used to control the emissivity of the backside of the sensors and probed by a LWIR commercial camera. Fundamental limits, noise, and band conversion efficiency were analyzed, indicating a great potential for this THz imaging technique to be further explored. Table 3 summarizes a comparison between the predicted and measured (where applicable) parameters of the fabricated sensors.

Table 3 shows that the metamaterial selective surface of the backside of sensor B, although not ideal, improved the efficiency while reducing the thermal time constant. The sensors’ efficiency can be further improved by designing both planar metamaterial surfaces, THz and IR sides, to be more selective in their respective bands. Due to the metamaterial configuration, the ground plane decouples the surfaces, making it possible to have completely different spectral responses on each side. In addition, multiband metamaterial structures can be designed to accommodate more than one THz illumination source as well as more than one readout in the IR band^37,42^. It can be observed in Fig. 8(a) that the spatial resolution of the sensor array is far from ideal. The arrays were fabricated to allow individual pixels to be studied. Therefore, the pixels were placed relatively far apart from each other to simplify the fabrication process. It follows that in order to improve spatial resolution, the space between pixels must be reduced. An attractive approach to achieve this is to use a membrane-like structure where the substrate, frame (Figs 2 and 6) would contain subarrays of pixels^20^. The speed of the sensors can be directly controlled by adjusting the width and length of the thermal insulator beams. For applications that demand higher frame rate, shorter and wider beams can be used to hold the sensing element in place, whereas for still images, thiner and longer beams should be employed.

The metamaterial-based THz-to-IR converter exhibits tremendous simplicity and has potential to achieve even higher efficiencies. Common MEMS materials and simple fabrication processes without readout electronics, as well as uncooled operation, open the possibility for this converter to be used as a simple attachment to commercial IR cameras to convert them into THz cameras without any additional modifications.

## Methods

### Simulation

**Figure 9:**
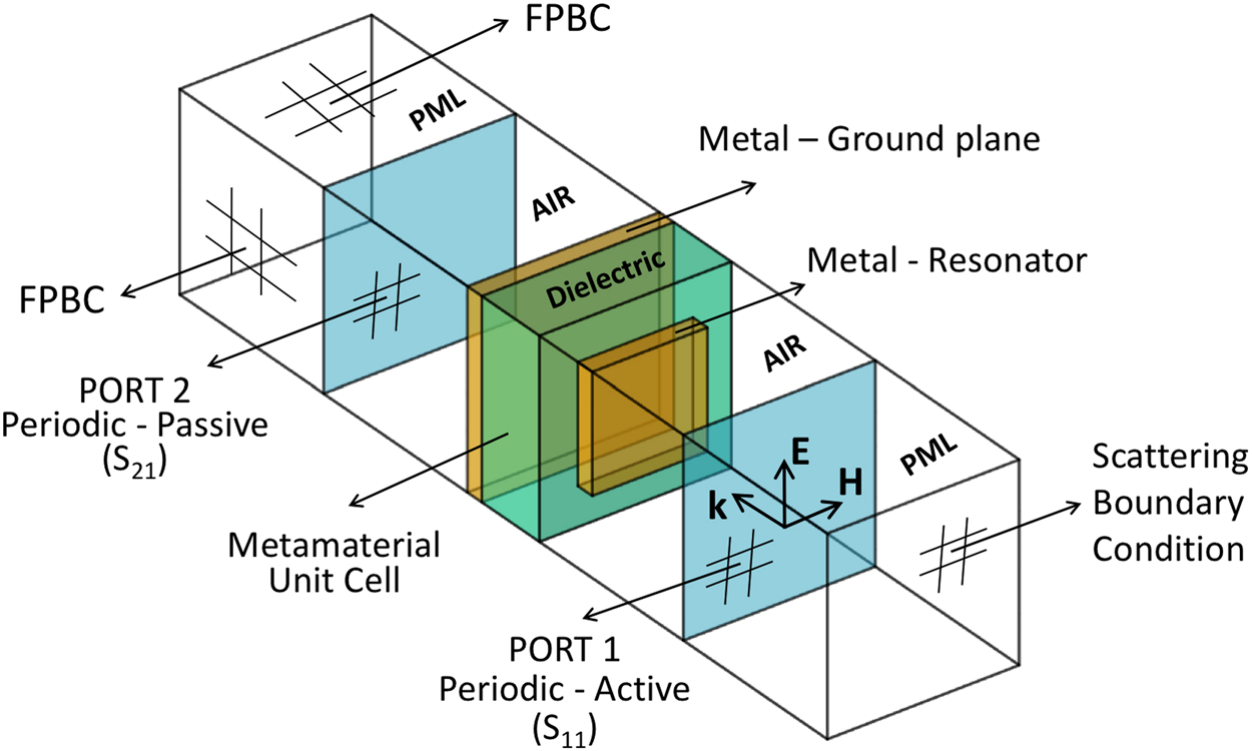
Metamaterial unit cell FE model. Schematic diagram of a two-port, PML-backed unit cell structure used to predict the frequency response of the THz metamaterials in COMSOL (Radio frequency module). FPBC stands for Floquet Periodic Boundary Condition, PML stands for Perfect Matched Layers while *S*_*11*_ and *S*_*21*_ are reflection and transmission scattering parameters.

The metamaterial structure was simulated using COMSOL Multiphysics, RF module where a two-port configuration as shown in Fig. 9 was used.

The metamaterial unit cell was sandwiched between air regions with active and passive ports as indicated in Fig. 9. This combination was further sandwiched between perfectly matched layers (PML) with scattering boundary conditions to assure nothing would reflect on the ports. Floquet periodic boundary conditions were used on the laterals. To improve the accuracy of the model, actual dimensions were used, including thicknesses of the metallic layers. Material properties were taken from Table 1, and no adjustable parameter was used for the RF simulation. A free triangular mesh was placed on the surface of the dielectric where the resonator sits and then extruded throughout all domains. The mesh element size was kept much smaller than the shortest wavelength on the E–H plane and more than 10 elements per domain on the k plane. A perpendicular plane wave is sent through the active port 1 and the scattering parameters *S*_*11*_ and *S*_*21*_ were extracted over the range of frequencies of interest. Reflectivity and transmissivity are given by |*S*_*11*_|^2^ and |*S*_*21*_|^2^, respectively. Since second-order scattering for such subwavelength structures is negligible and *S*_*21*_ is near zero, absorptivity of the metamaterial is obtained directly by 1 − |*S*_*11*_|^2^. To corroborate the latter statement, absorptivity was also obtained by integrating the resistive heat loss over the unit cell volume and normalized by the incident power.

**Figure 10:**
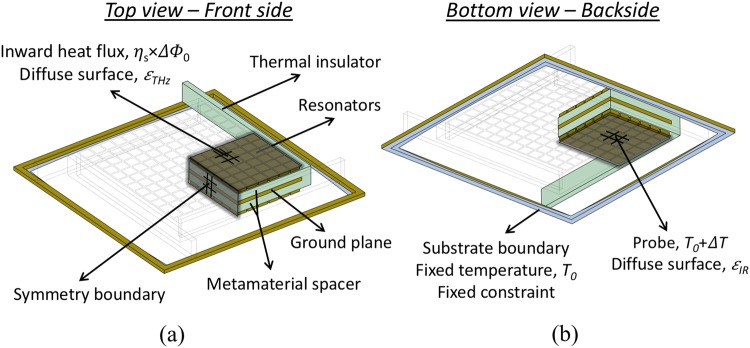
THz-to-IR sensor FE model. Schematic diagram of the front side (**a**) and backside (**b**) of one quarter of one pixel, used to estimate the figures of merit of sensor B in COMSOL (Heat Transfer and Surface-to-Surface Radiation Modules). The vertical dimension is exaggerated for visual clarity. For sensor A, the geometry was adapted accordingly.

The complete sensor structure (pixel) was also simulated using COMSOL, using solid mechanics and heat transfer in solids modules. Taking advantage of the axial symmetry in the **z** direction only one quarter of the sensor (shown in Fig. 10) was simulated in order to reduce the computational load.

Symmetry boundary conditions were applied to the surfaces on the cutting planes (in order to allow the thermal properties to be correctly calculated). All parts were considered linear elastic materials and were free to move except for the substrate frame. The frame was set as a fixed constraint, since it rests on the silicon substrate in the actual implementation of the sensor. The same frame was also set as a fixed temperature domain, representing the substrate heat sink, fixed at the ambient temperature, assumed to be 300 K for all simulations. This approximation was possible because the frame’s thermal mass in much larger than that of the detector.

The front side of the sensor was set as an inward heat flux, representing THz radiation the absorbed by the metamaterial structure. Meshing was performed in the same manner, identical to that of the metamaterial unit cell. Time dependent simulations were performed to determine both the responsivity and the thermal time constant of the sensor.

### Fabrication

**Figure 11:**
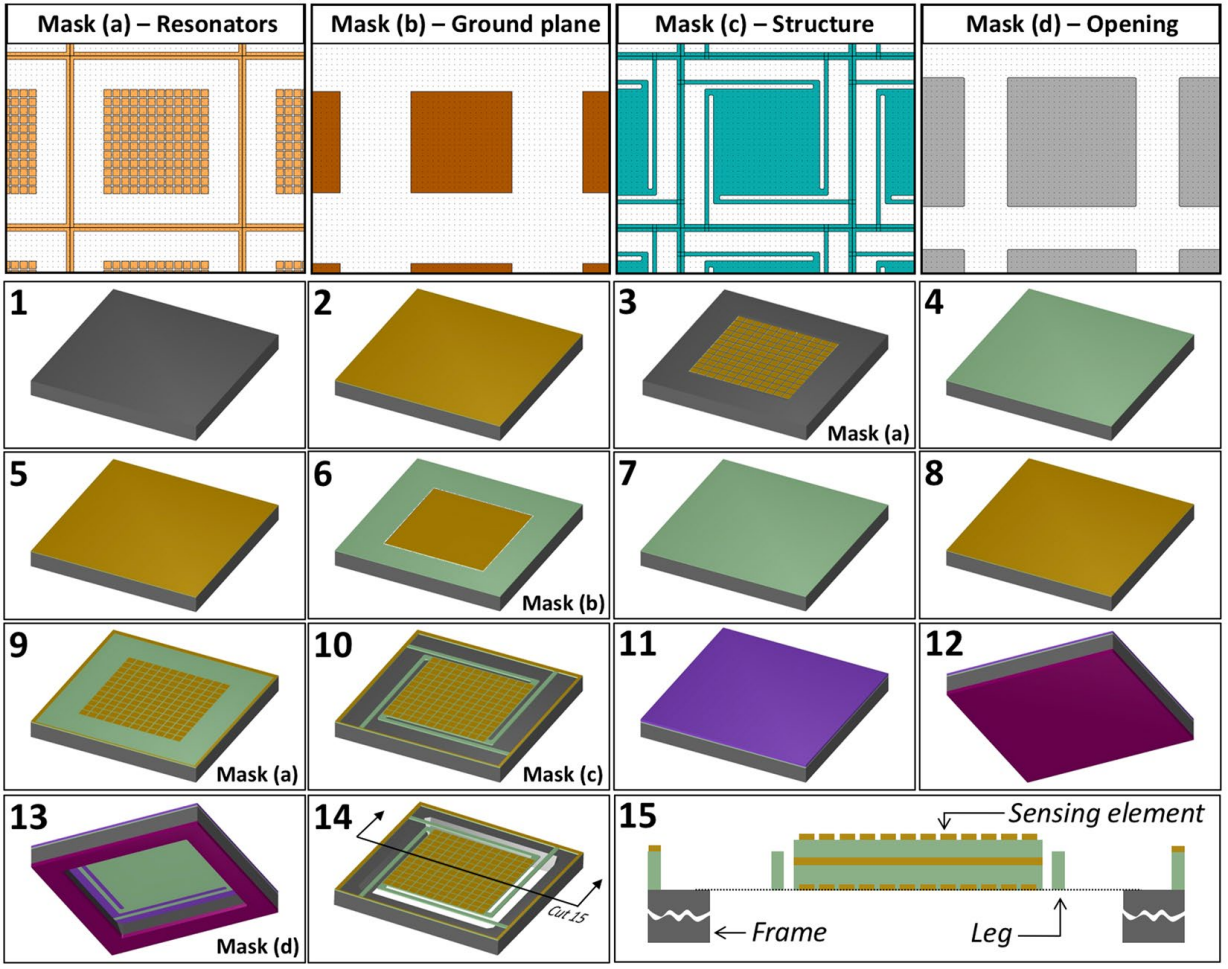
THz to IR FPA microfabrication steps. Fabrication sequence used for sensor B: (1) Si substrate, (2) backside resonator Al deposition, (3) backside resonator Al patterning, (4) backside SiO_x_ spacer deposition, (5) ground plane Al deposition, (6) ground plane Al patterning, (7) front side SiO_x_ spacer deposition, (8) front side resonator Al deposition, (9) front side resonator Al patterning, (10) sensor structure patterning, (11) front side protection, (12) back side protection, (13) back side substrate removal, (14) protection removal – sensor release. A cross section of the sensor after fabrication is shown in (15). For sensor A, steps (2) to (5) are not executed. The color codes for the materials are: Grey – Silicon; Golden Yellow – Aluminum; Green – Silicon oxide; Purple – SPR-220-7 photoresist (top side); and Wine – SPR-220-7 photoresist (bottom side).

Fabrication of the sensors was performed using standard MEMS microfabrication techniques. The photomasks layouts were designed using the application L-Edit from MEMSPro and sent out for fabrication. In case of both sensors, only four masks were necessary to pattern the resonators (a), ground plane (b), structure (c) and opening (d). The fabrication sequence for sensor B is shown in Fig. 11 and it can be described as follows.

Starting from a double-sided-polished 300 μm thick, p-type Si substrate with resistivity of 20 Ω.cm (1), a 90 nm of Al was deposited using a e-beam VE-240 Thermionics Lab metal evaporator (2). The bottom resonators were patterned (3) by argon (Ar) sputter-etch on an Oxford 100 reactive ion etcher (RIE), using mask (a). Next, 1.1 μm of SiO_x_ was deposited (4) using an Oxford 100 Plasma Enhanced Chemical Vapor Deposition (PECVD) instrument. The plasma power was set to 50 W at a chamber pressure of 50 mTorr, in which SiH_4_ and N_2_O flows were set as 20 and 100 sccm, respectively, and the substrate was kept at 300 °C. On top of that, 90 nm of Al was deposited, forming the ground plane for both metamaterial structures (5) and patterned (6) using mask (b). The second 1.1 μm SiOx layer was then deposited (7), followed by the final 90 nm Al layer (8). The top resonator layer was patterned (9) using mask (a), and the sensor structure was patterned (10) using mask (c), both by Ar sputter etch. The front side of the wafer was protected with a 7 μm thick layer of photoresist SPR 220-7, and baked for four hours at 90 °C (11). The bottom of the wafer was coated with a 7 μm thick layer of the same photoresist (12), and subsequently patterned with mask (d). The openings were etched from the bottom using Bosch Si etch on an Oxford 100 RIE (13). The devices were released with oxygen plasma on a barrel etcher IoN Wave 10 PVA for 10 minutes (14). Figure 11 box 15 shows the cross section of one pixel. Sensor A fabrication was similar, except that steps 2–4 were not executed and the Al thicknesses were different (Table 1). One important feature of MEMS fabrication is the residual stress, present in most MEMS free-standing structures. In the case of sensors A and B, the residual stress after release the structure (step 14 in Fig. 11) is not significant. The PECVD-grown silicon-rich silicon oxide (SiO_x_) films used in our sensors exhibit significantly less stress than the stoichiometric silicon dioxide (SiO_2_) layers. The numbers are available in a previous publication^50^. The aluminum influence is almost canceled by the fact that the dielectric is sandwiched between two aluminum layers with similar thicknesses. The bow caused on the pixel membranes is negligible as it can be seen in Fig. 6(a).

### Measurements

The spectral properties of the metamaterial structures were characterized using a Thermo-Nicolet Nexus 870 Fourier Transform Infrared Spectrometer (FTIR) with a globar source fitted with a PIKE Technologies MappIR accessory that allows for automatic transmissivity and reflectivity measurements. A gold coated Si wafer was used to establish the background for reflectivity measurements.

The THz-to-IR sensor was measured using a setup similar to the one shown in Fig. 1, except the QCL illumination source was directly focused on the FPA under test. Customized quantum cascade lasers (4.75 THz fabricated by LongWave Photonics Inc. and 3.8 THz fabricated by the Canadian National Research Council) were held at around 20 K by a closed cycle helium refrigerator, and controlled by a AVETECH laser diode driver model AVO-6HF-NPSB. Appropriate voltages were kept constant while pulse frequency was varied to control the average output power of the laser. An Agilent 33250A function generator was used to electronically gate the laser at lower frequencies. Responsivity was measured by changing the QCL power and recording the pixel temperature difference: We used a FLIR LWIR T1030sc camera controlled by a computer running FLIR ResearchIR software. The frequency response of the sensor was measured by varying the gate frequency from 0.1 to 30 Hz, and recording the temperature difference of the sensor using the same arrangement. The QCL power incident on the sensors under test was measured by a calibrated THz power meter. The instrument was comprised of the pyroelectric detector THZ-20 from Stanz – und LaserTechnik and a current pre-amplifier from the same company. The settings provided a response of 58.8 V/W, mostly flat between 1 and 5 THz. A Stanford Research Systems lock-in amplifier, model SR 850DSP was used to read the output of the pre-amplifier.

## Data Availability

The datasets generated during and/or analyzed during the current study are available from the corresponding author on reasonable request.
